# Health-Related Quality of Life Following Treatment for Testicular Cancer: A Qualitative Systematic Review

**DOI:** 10.1177/15579883251333619

**Published:** 2025-05-15

**Authors:** Louis Fox, Charlotte Moss, Tobias Gregor Hauser, Isolt Reardon, Netty Kinsella, Walter Cazzaniga, Mieke Van Hemelrijck, David Nicol

**Affiliations:** 1 Centre for Cancer, Society, and Public Health, King’s College London, UK; 2 Department of Urology, Barmherzige Brueder Hospital Munich, Germany; 3 Urology Unit, The Royal Marsden NHS Foundation Trust, London, UK

**Keywords:** testicular cancer, qualitative research, health-related quality of life, body image, social support

## Abstract

Testicular cancer (TCa) can precipitate ongoing psychosocial/physical morbidity post-treatment, despite high rates of cure. We conducted a systematic review to synthesise three decades of primary qualitative research on health-related quality of life (HRQoL) to inform the design of supportive care pathways. We queried MEDLINE/PubMed, PsycINFO, CINAHL, and Web of Science for all qualitative studies published up to 26 October 2022 examining issues relevant to HRQoL in post-treatment TCa patients. Two independent reviewers appraised included studies for methodological quality using established guidance for evidence-based public health policy and analysed findings via thematic synthesis. Studies were analytically weighted by study quality. Studies were evaluated using GRADE-CERQual to produce confidence levels in findings. We included 18 studies, comprising 387 participants. Seven studies were graded high quality, eight medium quality, and three low quality. Emergent analytical themes were: (a) Residual psychological injury; (b) Information deficits and unnecessary anxiety; (c) Thwarted life trajectory; (d) Social disruption; (e) Undermined youth; (f) Health service abandonment; (g) Long-term sexual problems; and (h) Maladaptive coping and post-traumatic stress. All findings were deemed ‘high confidence’, except theme (g), which was of ‘moderate confidence’. A limitation of the review was the relative dominance of Anglophone countries in the included studies. Some men treated for TCa are at risk of a range of post-treatment HRQoL issues, particularly psychosocial issues. Some individuals may be more at risk than others, based on specific personality traits, access to accurate and complete information about treatment recovery, and individual coping responses.

## Background

Testicular cancer (TCa) is one of the most common cancers amongst younger males and approximately 74,000 cases are reported per year globally, with population-adjusted incidence rates continuing to rise (Huang et al., 2022). Data from high-resourced regions such as those in Europe and North America demonstrate that TCa has, in recent decades, become a highly survivable disease (Cheng et al., 2018; Ehrlich et al., 2015).

Beyond these statistics, it is increasingly recognised that young men who receive treatment for TCa may experience challenges to their health-related quality of life (HRQoL), including both physical and psychosocial problems which may persist beyond typical follow-up and discharge (Cappuccio et al., 2018; Mercieca-Bebber et al., 2021; Patrikidou et al., 2023). While tools exist for quantifying HRQoL issues in TCa (e.g., Hoyt et al., 2013; Sztankay et al., 2018), recent analyses have identified shortcomings in these tools when subjected to comprehensive scrutiny of their psychometric attributes, including their content validity (i.e., whether a measure comprehensively measures all issues that it should) (Dincer et al., 2021; Rovito et al., 2021). Recent work undertaken by members of this group found that many TCa patients who are in the post-treatment phase find that existing patient-reported outcome measures omit questions that are important to them (Cazzaniga et al., 2023). Such findings undermine attempts to consolidate our understanding of the issues faced by this patient cohort after treatment (e.g., Mercieca-Bebber et al., 2021), given that limitations in tools unavoidably translate into limitations in findings.

This systematic review was undertaken as part of a wider prospective study (DISTANCE) that aims to consolidate our understanding of these post-treatment HRQoL issues and how they might be addressed in clinical practice, based on a broad analysis of all available quantitative and qualitative studies relevant to post-treatment HRQoL issues in TCa patients. To date, three decades of primary qualitative findings have not yet been synthesised, are often disparate, and can be challenging to interpret in terms of day-to-day considerations in clinical practice. The post-treatment HRQoL experiences of TCa patients have therefore largely remained clinical anecdotes which are of limited use. Hence, in our approach to this review, we aimed to close this gap and inform uro-oncology clinicians by producing formalised, yet ontologically open-ended empirical evidence based on the numerous existing qualitative studies examining post-treatment HRQoL issues in TCa patients.

## Methods

Methods are reported in accordance with the Enhancing Transparency in Reporting the Synthesis of Qualitative Research (ENTREQ) statement (Tong et al., 2012). The review was not preregistered, and no ethics approval was sought, as the original objective related primarily to the wider DISTANCE study. Consequently, this synthesis was not pre-planned. However, an appraisal of the search strategy used for DISTANCE indicated that a comprehensive qualitative evidence synthesis was feasible.

The research questions for the synthesis were:

Research question 1: What are the transferable insights that can be gained from studies examining experiences of men treated for TCa that are relevant to their HRQoL?
Research question 2: What are the relationships between these insights and: (a) treatment(s) received; (b) life phases; (c) time since treatment; and (d) personal individual differences between patients?

### Search Strategy

Using a pre-planned (i.e., non-iterative) search strategy executed as part of the wider DISTANCE study, we queried MEDLINE/PubMed, PsycINFO, CINAHL, and Web of Science on 26 October 2022 (for full search strategy, see Supplemental Material 1). The databases were chosen due to their coverage of three key domains of medical practice, psychosocial phenomena, and nursing practice.

### Screening and Inclusion

All retrieved records were independently screened for inclusion by two reviewers (LF and IR). Broadly, the inclusion criteria were:

(1) any peer-reviewed original research study;
(2) of qualitative design;
(3) utilising primary or secondary data analysis;
(4) which examined the experiences of adult individuals diagnosed with TCa;
(5) in which the research aim, objective, or primary or secondary research question(s) place those experiences in the context of a TCa diagnosis of either the participant, or someone known to the participant (full inclusion/exclusion criteria contained in Supplemental Material 2).

Records were screened for inclusion/exclusion first by review of their titles, then their abstracts, and then full-text articles. Disagreements between reviewers about inclusion were resolved via reasoned discussion. A third reviewer was to be involved in case of impasse but was ultimately not required.

### Quality Appraisal

Quality appraisal of all included studies was undertaken to enable analytical weighting of study insights. At the time of undertaking this review, there were no universally agreed criteria for qualitative study appraisal (the CAMELOT tool [Munthe-Kaas et al., 2024], which addresses this issue, was published in June 2024). The Critical Appraisal Skills Programme (CASP) tool (Long et al., 2020) is most used; however, this tool was designed (and indeed is only intended to be used) as a pedagogical instrument and was considered less suitable for our aims. Rather, we opted to use criteria outlined by guidance [PMG4] of the Centre for Public Health Excellence of the UK’s National Institute for Health and Care Excellence (NICE), which informs public health policy decision making in the UK (National Institute for Health and Care Excellence, 2012). The PMG4 guideline on qualitative study appraisal is an amalgamation of three other quality appraisal checklists as a pragmatic response to a lack of a universally agreed tool. On review, we judged it to possess stronger analytical coverage of the CASP tool and better suited to our objectives.

All included studies were independently critically appraised by two reviewers (LF and CM) in accordance with the criteria outlined in NICE [PMG4]. Disagreements were identified per individual appraisal criterion and were categorised as either ‘partial disagree’ (one neutral appraisal and one positive/negative appraisal) or ‘opposing disagree’ (one positive appraisal and one negative appraisal). Each disagreement was resolved via discussion. Following appraisal of all criteria, each study was categorised as ‘High quality’, ‘Medium quality’, or ‘Low quality’ in accordance with the approach outlined in NICE [PMG4].

### Data Extraction and Synthesis

We undertook a thematic synthesis of the data based on the method outlined by Thomas and Harden (2008), using NVivo 14 (Lumivero). Thematic synthesis was deemed an appropriate approach to produce the type of generalised insights required for our applied setting. The analytical approach was modified to enable analytical weighting of study insights according to methodological quality of each study, using the method proposed by Long et al. (2020). In brief, the method stipulates that insights taken from high-quality studies are analysed first and comprise the primary coding index, which is inductively constructed from these studies only. Insights from the medium-quality studies are then integrated into this coding index, with new codes created from these studies only if the insight provided is deemed to be highly relevant and adjunctive to existing codes, and/or consistent with/integral to an emergent thematic pattern. Insights from low-quality studies are then used only to corroborate insights obtained from higher-quality studies (i.e., they are not used to produce any additional new codes). In this way, all data codes underpinning the final analytical themes are based on data that has a robust methodological grounding.

Text contained in the *Results* and *Discussion* sections of the included articles was independently coded by two reviewers (LF and CM) in line with the above approach. Pragmatic coding was undertaken, in which segments of text were deductively identified in accordance with their relevance to the above research question(s). Codes were then produced inductively from the data, independently by both reviewers, and integrated into a single code structure based on a code-by-code discussion between reviewers, enabling the triangulation of descriptive and, where feasible, conceptual themes.

First, high-quality studies were used to produce a primary coding structure as described above (with a review meeting after coding the first study to ensure methodological alignment). Where possible, all codes were labelled with attributes according to the circumstances they pertained to (e.g., what treatment had been received; what the relative age of the patient was; whether the patient was in education; whether the patient had a family; specific personality traits etc.). Then, LF and CM met to: (a) integrate the two coding structures into one via a code-by-code discussion of all codes; (b) refine the names and definitions of resultant unified codes based on agreed conceptual definitions; and (c) begin to develop descriptive themes. Then, medium-quality studies were coded into this thematic structure as described above; and following this, LF and CM met once again to integrate the newly semi-divergent thematic structures into one, similarly to above; and to refine the descriptive themes. Low-quality studies were then deductively coded in accordance with the already existing code structure.

LF and CM then had a series of discussions of the data to ensure alignment on the analytical structure of the emerging themes and associated sub-themes and to develop integrated analytical themes based on the logical, conceptual relationships or overlap between the descriptive themes and the properties of the different data insights (i.e., pertaining to what specific type of patient or treatment), in terms of the known circumstances of the patients that produced that data.

### Evaluation of Findings

Once the synthesis was complete, the individual review findings (i.e., the analytical themes), and the data underpinning them, were retroactively evaluated by three reviewers (TGH, CM, and LF) to ascertain a confidence level of the validity of each individual finding, using the GRADE-CERQual approach outlined by Lewin et al. (2018). GRADE-CERQual is a methodical way of producing a concise summary of key findings; the reviewers’ confidence in each finding based on the potential for methodological issues to be underlying the finding; and concise summaries of methodological concerns (if any) that may undermine confidence in each finding.

## Results

**Figure 1.**
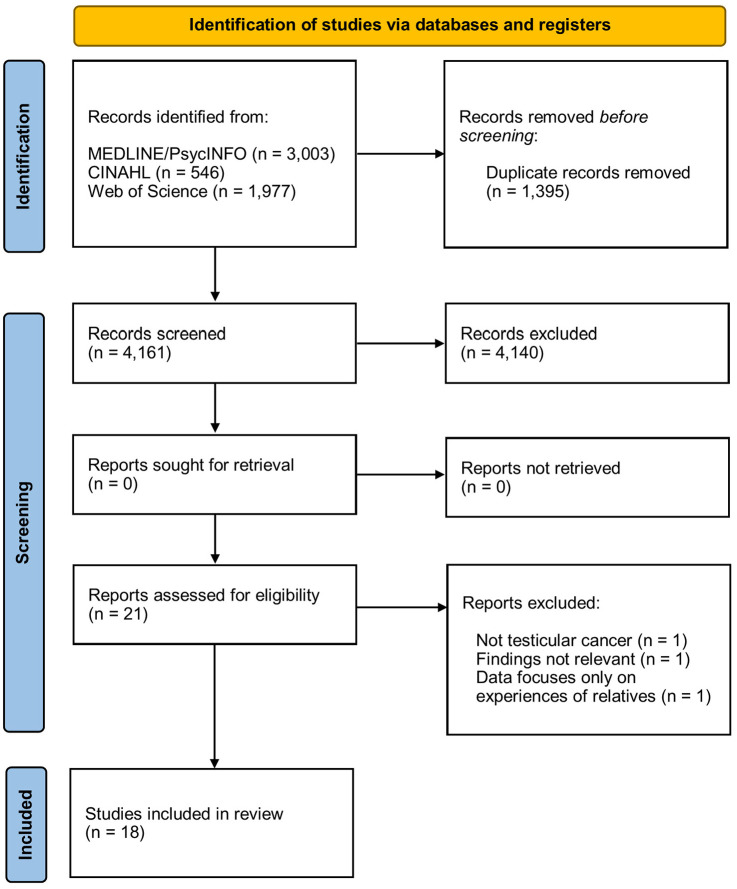
PRISMA Flow Diagram of Inclusion Process

The wider search (for the wider DISTANCE project) returned 4,161 studies, of which 18 were included for this analysis, representing 387 individual participant perspectives (Figure 1) (Achille et al., 2006; Brodsky, 1995, 1999; Carpentier et al., 2011; Chapple & McPherson, 2004; Gupta et al., 2020; Gurevich et al., 2004; Håland et al., 2017; Hoyt et al., 2016; Johansson et al., 1992; Kristjanson et al., 2006; Macdonald, 2001; Matheson et al., 2016; Petrella et al., 2020; Robitaille, 2009; Saab et al., 2014; Shen et al., 2016; Sheppard & Wylie, 2001). Characteristics of included studies are shown in Table 1. During the critical appraisal process, there was full agreement between LF and CM on 77% of the 270 individual appraisal decisions (a further 14% and 9% resulted in partial or opposing disagreement, respectively) (see Supplemental Material 3). Once disagreements were resolved, seven studies were graded high quality (Achille et al., 2006; Brodsky, 1995; Kristjanson et al., 2006; Matheson et al., 2016; Petrella et al., 2020; Saab et al., 2014; Shen et al., 2016), eight studies were graded medium quality (Brodsky, 1999; Carpentier et al., 2011; Chapple & McPherson, 2004; Gupta et al., 2020; Gurevich et al., 2004; Hoyt et al., 2016; Macdonald, 2001; Robitaille, 2009), and three studies were graded low quality (Håland et al., 2017; Johansson et al., 1992; Sheppard & Wylie, 2001). Of the 387 individual participant perspectives, 319 were from high- or medium-quality studies, and 131 were from high-quality studies.

**Table 1.** Characteristics of Included Studies.

Author(s) and year	Country	Concept area	Data collection method	Analysis method	Sampling method	No. of participants	Time period since treatment (months)	Study quality
Matheson et al. (2016)	UK	Appraisal and coping	Semi-structured interviews (at two time points)	Grounded theory	Convenience	18	0–12	High
Saab et al. (2014)	Lebanon	Psychosexual impact	Semi-structured interviews / observational field notes	Interpretive phenomenology	Purposive	8	>24	High
Kristjanson et al. (2006)	Australia	Support needs and coping strategies	Semi-structured interviews	Content analysis	Purposive	32	0–24	High
Brodsky (1995)	USA	Effects on psychological self	Open-ended interviews	Thematic analysis	Snowball	11	>24	High
Achille et al. (2006)	Canada	Barriers and facilitators to sperm banking	Semi-structured interviews	Framework analysis	Purposive	38 (incl. 18 clinicians)	>24	High
Petrella et al. (2020)	Canada	Experiences of survivorship and perspectives on sport-based supportive care	Semi-structured interviews	Thematic analysis	Convenience	11	>24	High
Shen et al. (2016)	Canada	Transition to survivorship	Semi-structured interviews / focus groups	Content analysis	Convenience	13	6–24	High
Gupta et al. (2020)	USA	Financial stress	Semi-structured interviews	Non-specific mixed analysis (secondary data analysis)	Convenience	20	>24	Medium
Chapple and McPherson (2004)	UK	Prosthesis decision-making	Semi-structured interviews	Thematic analysis	Maximum variation	45	>12	Medium
Carpentier et al. (2011)	USA	Psychosexual impact	Semi-structured interviews	Thematic analysis	Purposive	21	>3	Medium
Gurevich et al. (2004)	Canada	Gender identity	Semi-structured interviews	Thematic decomposition	Convenience	40	>3	Medium
Robitaille (2009)	Canada	Post-traumatic stress and/or growth	Semi-structured interviews	Non-specific mixed analysis (secondary data analysis)	Purposive	12	>12	Medium
Macdonald (2001)	Canada	Impacts on self-identity	Semi-structured interviews	Grounded theory	Purposive (theoretical)	18	>3	Medium
Hoyt et al. (2016)	USA	Goal navigation and psychological adjustment	Semi-structured interviews	Thematic analysis	Purposive	21	>12	Medium
Brodsky (1999)	USA	Psychological impact	Semi-structured interviews / observational field notes	Non-specific ethnographic method	Convenience	11	>24	Medium
Sheppard and Wylie (2001)	UK	Psychosexual impact	Semi-structured interviews	Thematic analysis	Convenience	6	>0	Low
Håland et al. (2017)	Norway	Appraisal and coping	Semi-structured interviews	Systematic text condensation	Convenience	5	0–48, not clearly specified	Low
Johansson et al. (1992)	Sweden	Aspects of follow-up care	Structured interviews	Not specified	Convenience	39	>12	Low

Analytical themes and associated sub-themes are presented below in list form for conciseness and ease of interpretation/application (for supporting data exemplars, please refer to Supplemental Material 4). Due to the study weighting procedure, the insights presented below mostly represent the seven highest quality studies, and then to a lesser extent, the eight medium-quality studies. Low-quality studies were not used to produce the below insights (only to corroborate them).

### Analytical Theme 1: Residual Psychological Injury

While curative treatment is a precondition for recovery from psychological injuries, it does not precipitate such recovery in all cases.

- Information deficit can compound psychological issues.
   ○ Information deficit related to oncological prospects of TCa patients can cause, or exacerbate, ongoing anxiety post-treatment.
   ○ Information deficit related to what to expect in the post-treatment recovery phase (and transition to survivorship) may produce psychologically destabilising uncertainty, which may feed into fear and anxiety.
- Damage to fertility increases the likelihood of present or future psychological injury.
   ○ Relatively older men (in their mid-to-late twenties, or in their thirties) who are in relationships are more likely to suffer immediate psychological injury.
   ○ Relatively younger men (adolescents and early twenties) who have not much considered child-rearing may be at less risk for immediate psychological injury, but may experience future psychological injury not any less serious.
   ○ The immediate physical threat of a yet-to-be-treated cancer may cause patients to attempt to relegate fertility concerns as a low priority and not act upon them (by sperm banking) until it is too late. Certain behaviour by the clinical team has the potential to encourage this de-prioritisation.
- Surgical treatments can produce long-term body image issues.
  ○ Some men will experience body image issues related to orchiectomy. However, this may not be as prevalent in this treatment population as might be commonly assumed.
    ▪ Such body image issues may be likely to arise as a function of the extent that the individual perceives their testicle as emblematic of an idealised masculinity.
  ○ Threats to body image are relatively more salient for many men who have visible surgical scars; this is due to their outward appearance instigating cancer disclosure to others.

### Analytical Theme 2: Information Deficits and Unnecessary Anxiety

An information deficit regarding oncological risk, or what to expect from the recovery phase, can put recovering TCa patients at higher risk of anxiety; and produce a psychologically destabilising sense of uncertainty.

- Information deficit related to treatment recovery can produce a destabilising sense of uncertainty, which can thwart crucial normalisation processes post-treatment.
- Information deficit related to the recurrence rate can lead to unnecessary anxiety.
- The presence of a supportive person (e.g., family member, partner, friend) at appointments may make a materially positive difference to how much information a patient is able to absorb.

### Analytical Theme 3: Thwarted Life Trajectory

TCa can be strongly disruptive to a patient’s preconceived ideas of their life trajectory.

- Disruptions to social life trajectory are possible, particularly for relatively younger patients.
- Disruptions to educational and/or professional life trajectories are possible, particularly for relatively younger patients. In adolescent patients, this may result in increased dependence on parents and subsequently a lack of young adult autonomy.
- Disruptions to prospective family life are possible (i.e., starting a family), particularly for those hoping to start a family soon.

### Analytical Theme 4: Social Disruption

Important social dynamics between the patient and others in their life can be disrupted by the TCa experience.

- Individuals in long-term committed relationships are relatively less likely to report significant negative disruption to relationships than individuals in less established, short-term relationships. Those in less established, shorter-term relationships may experience negative disruption to those relationships.
- Patients who are not in a relationship are likely to experience anxiety about prospective romantic relationships. Such anxieties can be particularly salient for men who have had orchiectomy, but it can apply to any patients, regardless of treatment type.
- The cancer event can cause alienation of the patient from certain friends, who, for various possible reasons, may lack the capability to engage with the patient’s experience.
- Patients may find workplace peers and superiors to be either accommodating and supportive, or troublesome and/or actively unsupportive, depending on their workplace context. The latter situation produces additional stress and anxiety.
- Patients with their own families may psychologically suffer due to a perceived inability to perform self-imposed family roles, as a result of maladaptive coping in response to their experience.

### Analytical Theme 5: Undermined Youth

Patients’ sense of youthful vitality and sense of young-adult autonomy can be undermined by TCa.

- In some adolescent patients, a lack of professional/financial independence can make a patient re-dependent upon their parents; particularly as parents are likely to step into a carer role during a patient’s treatment, hence providing a pathway to re-dependence when combined with the temporary cessation of progress in the patient’s education or professional development.
- Engagement with hobbies that were enjoyed prior to diagnosis can act as anchors of continuity, and hence be an effective restoration method, contributing to normalisation processes.
- Similarly to above, engaging in physical activity can provide a vehicle for a patient to reconnect with and taking back control of their body and mind.
  ○ Physical activity may provide a useful psychologically therapeutic ‘trojan horse’ in the cases of emotionally closed patients who might resist other methods of psychological support or therapy.
- For some patients, in-person follow-up appointments involving having to go to a hospital can physically and psychologically interfere with important normalisation processes.

### Analytical Theme 6: Health Service Abandonment

Some patients experience feelings of health service abandonment following completion of treatment and follow-up.

- Feelings of health service abandonment following discharge can precede – and perhaps precipitate – the onset of emotional crises linked to the trauma of the patient’s experience with TCa.
- The removal of ongoing support is experienced as an acute psychological event by some patients, and this may be particularly true for patients with residual issues (physical or psychological).

### Analytical Theme 7: Long-Term Sexual Problems

Patients may experience long-term disruption to their sex life.

- Some patients may feel unable to engage in sex due to physical problems such as pain or erectile issues.
- Some patients may experience powerful anxiety in anticipation of sexual situations.
- Some patients may experience decreased physical pleasure during sexual intercourse.

### Analytical Theme 8: Maladaptive Coping and Post-Traumatic Stress

Individuals with a recent history of TCa treatment are at risk of maladaptive coping responses and emergent post-traumatic stress after being discharged from care.

- Post-traumatic stress experiences may manifest sometime after routine follow-up and discharge.
- The risk of a general maladaptive coping response may be higher if:
  ○ The patient is not in a relationship and lacks reliable peers for support.
  ○ The patient is relatively less able to be cognitively flexible about life goals not yet achieved, which are important to them.
  ○ The patient has personality characteristics that inflexibly conform to traditional cultural norms around gender.
    ▪ The above may be compounded by treatment with orchiectomy.
    ▪ The above may be compounded by treatments impacting fertility (if the patient is childless).
  ○ The patient has lacked an opportunity, throughout the provision of their care, to have the mental space to reflect on their situation in a supportive environment.
  ○ The patient experiences the transition from follow-up to discharge (and beyond) as a psychologically disruptive event.

The outcome of the GRADE-CERQual evaluation of the synthesis findings is presented in Table 2, which displays the confidence level of each finding as per the GRADE-CERQual criteria. As per GRADE-CERQual criteria, there was deemed to be ‘high confidence’ in seven of the eight core findings (i.e., the analytical themes). One of the core findings, ‘Health service abandonment’, was deemed to be of ‘moderate confidence’. We had some ‘minor’ concerns and some ‘moderate’ concerns regarding the criteria of *coherence*, *adequacy*, or *relevance* of the data, for some of the findings. We had no concerns regarding the *methodological limitations* criterion with regard to any of the findings.

**Table 2.** GRADE-CERQual Assessment of Qualitative Evidence Across Eight Core Findings.

Summary of review findings	Studies contributing to the review findings	Methodological limitations	Coherence	Adequacy	Relevance	CERQual assessment of confidence in the evidence	Explanation of CERQual assessment
*Residual psychological injury*: While curative treatment is a precondition for recovery from psychological injuries, it does not precipitate such recovery in all cases.	Håland et al., Matheson et al., Hoyt et al., Brodsky, Shen et al., Saab et al., Chapple and McPherson, Kristjanson et al., Carpentier et al., Sheppard and Wylie, Brodsky, Gurevich et al., Petrella et al., Robitaille et al., Macdonald	No or very minor concerns	No or very minor concerns	No or very minor concerns	No or very minor concerns	High confidence	No concerns.
*Information deficits and unnecessary anxiety*: An information deficit regarding oncological risk, and what to expect from the recovery phase, can place recovering testicular cancer patients at higher risk of anxiety, and also produce a psychologically destabilising sense of uncertainty.	Håland et al., Matheson et al., Brodsky, Shen et al., Kristjanson et al., Carpentier et al., Sheppard and Wylie, Gurevich et al., Achille et al., Petrella et al., Robitaille et al., Macdonald	No or very minor concerns	Minor concerns	No or very minor concerns	No or very minor concerns	High confidence	Whereas clear evidence underlined the connection between disease-related anxiety and information deficits regarding testicular cancer, the longer-term destabilising effect received relatively more limited coverage across reviewed studies.
*Thwarted life trajectory*: Testicular cancer can be strongly disruptive to a patient’s preconceived ideas of their life trajectory.	Gupta et al., Matheson et al., Hoyt et al., Brodsky, Saab et al., Kristjanson et al., Sheppard and Wylie, Brodsky, Gurevich et al., Petrella et al., Robitaille et al., Macdonald	No or very minor concerns	No or very minor concerns	No or very minor concerns	No or very minor concerns	High confidence	No concerns.
*Social disruption*: Important social dynamics between the patient and others in their life can be disrupted by the testicular cancer experience.	Matheson et al., Brodsky, Shen et al., Saab et al., Chapple and McPherson, Kristjanson et al., Carpentier et al., Sheppard and Wylie, Brodsky, Gurevich et al., Petrella et al., Robitaille et al., Macdonald	No or very minor concerns	No or very minor concerns	No or very minor concerns	No or very minor concerns	High confidence	No concerns.
*Undermined youth*: Patients’ sense of youthful vitality and sense of young-adult autonomy can be undermined by testicular cancer.	Gupta et al., Matheson et al., Hoyt et al., Carpentier et al., Sheppard and Wylie, Gurevich et al., Petrella et al., Robitaille et al., Macdonald	No or very minor concerns	No or very minor concerns	No or very minor concerns	No or very minor concerns	High confidence	No concerns.
*Health service abandonment*: Some patients experience feelings of health service abandonment following completion of treatment and follow-up.	Brodsky, Petrella et al., Robitaille et al.	No or very minor concerns	Minor concerns	Moderate concerns	Minor concerns	Moderate confidence	Only limited data was available on this phenomenon as it has not been the *a priori* focus of data collection in any of the included studies. Since different aspects of subjective health service abandonment were raised by some, yet not the majority of individuals during interviews, its importance remains to be further investigated.
*Long-term sexual problems*: Patients may experience long-term disruption to their sex life.	Brodsky, Saab et al., Chapple and McPherson, Kristjanson et al., Carpentier et al., Sheppard and Wylie, Brodsky, Gurevich et al., Petrella et al., Macdonald	No or very minor concerns	Moderate concerns	No or very minor concerns	No or very minor concerns	High confidence	Moderate coherence concerns arose from the fact that information on long-term sexual problems and fear of those arising was somewhat inconsistent at an individual patient level. Given the extent of data covering different aspects of this phenomenon, the importance and general existence of this theme for some patients can however be stated with high confidence.
*Maladaptive coping and post-traumatic stress*: Individuals with a recent history of testicular cancer treatment are at risk of maladaptive coping responses, and emergent post-traumatic stress after being discharged from care.	Gupta et al., Håland et al., Matheson et al., Hoyt et al., Brodsky, Shen et al., Saab et al., Chapple and McPherson, Kristjanson et al., Sheppard and Wylie, Brodsky, Gurevich et al., Achille et al., Petrella et al., Robitaille et al., Macdonald	No or very minor concerns	No or very minor concerns	No or very minor concerns	No or very minor concerns	High confidence	No concerns.

## Discussion

The results of our review help to empirically codify some key HRQoL issues in TCa patients, which may be anecdotally familiar to many urology clinicians, and have consolidated key insights which are worthy of consideration in care pathways for TCa patients. The results show that numerous good quality qualitative studies demonstrate that TCa patients can experience residual psychological injuries; experience unnecessary anxiety due to information deficits; feel that their life trajectory has been thwarted; experience disruption to their friendships or short-term romantic relationships; experience undermined youthfulness and young-adult autonomy; experience feelings of health service abandonment following discharge from care; experience long-term (psycho)sexual problems; and in certain circumstances, engage in maladaptive coping responses or experience delayed-onset post-traumatic stress.

The evidence reported here is consistent with quantitative research on TCa survivors of >6 m indicating that HRQoL in TCa survivors post-treatment is most impacted by psychosocial, rather than physical, issues; and that while most patients experience only mild psychosocial morbidity, a smaller vulnerable subgroup demonstrate severe morbidity (Smith et al., 2016; Yamashita et al., 2021). The strength of qualitative approaches such as those represented here is that we can identify precisely what events and circumstances precipitate and govern HRQoL morbidity, which can produce informed solutions to enhance the supportive care pathway. A limitation of the qualitative approach is uncertainty regarding the actual incidence/prevalence of the issues reported. Hence, it is informative that existing quantitative findings align with the findings we report in our review, demonstrating that the issues outlined in our results are indeed likely to represent a considerable HRQoL burden in the TCa survivor population. It has been estimated that approximately one in five TCa survivors are, at any given time, experiencing moderate, severe, or extremely severe depression, anxiety, or stress, which is approximately 50% greater prevalence than comparable normative data (Smith et al., 2016). In particular, quantitative data has indicated that patients in receipt of both chemotherapy and retroperitoneal lymph node dissection (RPLND) together may be at heightened risk in terms of disruption to the life course and HRQoL (Yamashita et al., 2021). The data we reviewed were limited in this regard, as links to chemotherapy and RPLND were not made explicit in the reviewed data. However, there were many references to ‘recovery from treatment’, from which it can perhaps be deduced – considering the quantitative evidence – that this therapeutic regimen played a role in the HRQoL issues documented, in some cases.

While we were unable to expound upon appropriate supportive care solutions within the scope of this article (this will form the basis of future work), we can provide some cursory insights on supportive measures that may be useful for addressing HRQoL that incidentally emerged from our analysis of these qualitative studies:

1. Upfront, realistic, comprehensive information on what to expect from the recovery phase.
2. Support strategies acknowledging patriarchal influences on patients’ lived experiences of TCa, including evaluation of general psychological adaptability and support-seeking behaviour.
3. Pro-active sperm banking advice and protocols, which acknowledge that amid diagnosis, a patient may lack ‘headspace’ to make a decision about sperm banking by themselves.
4. Opportunities for patients to psychologically reflect and process their experience during their care, and sustained follow-up to monitor for manifestations of post-traumatic stress.
5. Innovative peer-support interventions acceptable to this relatively younger, male patient demographic (e.g., group physical activity signposting or digital solutions).

### Study Limitations

There were some limitations to this review. The methodology precludes the quantification of the relative burden of the reported issues across the TCa patient population. Furthermore, most (although not all) included studies were from the UK, USA, and Canada, which raises issues of cross-cultural transferability.

### Clinical Implications

This review consolidates qualitative insights that are crucial to providing comprehensive care for post-treatment TCa patients. The information reported in this review can be taken forward to assist in the update and development of existing and new HRQoL measurement tools for those affected by TCa and to enhance supportive care pathways based on the conclusions below.

## Conclusions

In conclusion, the existing literature identifies several potential key domains which could be addressed to maximise HRQoL outcomes in the post-treatment phase: (a) adequate information provision about what to expect post-treatment; (b) identification of – and appropriate support for – patients who do not have a robust social support network (such as being in a committed relationship); (c) opportunities for psychological reflection to facilitate some emotional processing of the experience (and longer-term monitoring to pick up cases of emotional and traumatic stress-related problems); (d) mitigation of the potential psychological shock of healthcare system discharge; (e) pro-active sperm banking advice which acknowledges the limited capacity of the patient to make this decision at the time it is required to be made; (f) encouragement, where appropriate, to engage in normalising activities such as pre-existing hobbies and physical activity; (g) counselling regarding life goals and transition to adulthood; and (h) psychosexual counselling.

## Supplemental Material

sj-docx-1-jmh-10.1177_15579883251333619 – Supplemental material for Health-Related Quality of Life Following Treatment for Testicular Cancer Supplemental material, sj-docx-1-jmh-10.1177_15579883251333619 for Health-Related Quality of Life Following Treatment for Testicular Cancer by Louis Fox, Charlotte Moss, Tobias Gregor Hauser, Isolt Reardon, Netty Kinsella, Walter Cazzaniga, Mieke Van Hemelrijck and David Nicol in American Journal of Men's Health

sj-docx-2-jmh-10.1177_15579883251333619 – Supplemental material for Health-Related Quality of Life Following Treatment for Testicular Cancer Supplemental material, sj-docx-2-jmh-10.1177_15579883251333619 for Health-Related Quality of Life Following Treatment for Testicular Cancer by Louis Fox, Charlotte Moss, Tobias Gregor Hauser, Isolt Reardon, Netty Kinsella, Walter Cazzaniga, Mieke Van Hemelrijck and David Nicol in American Journal of Men's Health

sj-pdf-5-jmh-10.1177_15579883251333619 – Supplemental material for Health-Related Quality of Life Following Treatment for Testicular Cancer Supplemental material, sj-pdf-5-jmh-10.1177_15579883251333619 for Health-Related Quality of Life Following Treatment for Testicular Cancer by Louis Fox, Charlotte Moss, Tobias Gregor Hauser, Isolt Reardon, Netty Kinsella, Walter Cazzaniga, Mieke Van Hemelrijck and David Nicol in American Journal of Men's Health

sj-pdf-6-jmh-10.1177_15579883251333619 – Supplemental material for Health-Related Quality of Life Following Treatment for Testicular Cancer Supplemental material, sj-pdf-6-jmh-10.1177_15579883251333619 for Health-Related Quality of Life Following Treatment for Testicular Cancer by Louis Fox, Charlotte Moss, Tobias Gregor Hauser, Isolt Reardon, Netty Kinsella, Walter Cazzaniga, Mieke Van Hemelrijck and David Nicol in American Journal of Men's Health

sj-xlsx-3-jmh-10.1177_15579883251333619 – Supplemental material for Health-Related Quality of Life Following Treatment for Testicular Cancer Supplemental material, sj-xlsx-3-jmh-10.1177_15579883251333619 for Health-Related Quality of Life Following Treatment for Testicular Cancer by Louis Fox, Charlotte Moss, Tobias Gregor Hauser, Isolt Reardon, Netty Kinsella, Walter Cazzaniga, Mieke Van Hemelrijck and David Nicol in American Journal of Men's Health

sj-xlsx-4-jmh-10.1177_15579883251333619 – Supplemental material for Health-Related Quality of Life Following Treatment for Testicular Cancer Supplemental material, sj-xlsx-4-jmh-10.1177_15579883251333619 for Health-Related Quality of Life Following Treatment for Testicular Cancer by Louis Fox, Charlotte Moss, Tobias Gregor Hauser, Isolt Reardon, Netty Kinsella, Walter Cazzaniga, Mieke Van Hemelrijck and David Nicol in American Journal of Men's Health
