# Supplementary material for: Health-Related Quality of Life Following Treatment for Testicular Cancer: A Qualitative Systematic Review
Source: Am J Mens Health. 2025 May 15;19(3):15579883251333619. doi: 10.1177/15579883251333619 (PMC12081962; doi:10.1177/15579883251333619)
Supplement: sj-pdf-5-jmh-10.1177_15579883251333619 – Supplemental material for Health-Related Quality of Life Following Treatment for Testicular Cancer [file sj-pdf-5-jmh-10.1177_15579883251333619.pdf]

# Supplementary Material 2

## Inclusion and exclusion criteria

| Inclusion criteria |
| --- |
| • Any peer-reviewed original research study of qualitative design, utilising primary or secondary data analysis, which examines the experiences of adult individuals diagnosed with testicular cancer, in which the research aim, objective, or primary or secondary research question(s) place those experiences in the context of a testicular cancer diagnosis of either the participant or someone known to the participant |
| **Exclusion criteria** |
| • Studies in which the entire sample is comprised of patients diagnosed less than 3 months prior to either enrolment or data collection<br>• Studies with samples that contain a mixture of cancer types – with the exception of studies where the data pertaining specifically to testicular cancer patients is relevant and can be isolated in the process of data extraction<br>• Studies of paediatric patients only<br>• Conference abstracts<br>• Reviews, protocols, case reports, commentaries, letters, and editorials<br>• Articles not in English |
