# Supplementary material for: Health-Related Quality of Life Following Treatment for Testicular Cancer: A Qualitative Systematic Review
Source: Am J Mens Health. 2025 May 15;19(3):15579883251333619. doi: 10.1177/15579883251333619 (PMC12081962; doi:10.1177/15579883251333619)
Supplement: sj-pdf-6-jmh-10.1177_15579883251333619 – Supplemental material for Health-Related Quality of Life Following Treatment for Testicular Cancer [file sj-pdf-6-jmh-10.1177_15579883251333619.pdf]

Supplementary Material 1

Search strategy

	CINAHL
1	TX (testicular cancer OR testicular neoplasms OR testis cancer OR testes cancer) AND TX (interview* OR focus group* OR ethnograph* OR qualitative research OR qualitative study OR qualitative analysis OR qualitative methods OR quality of life OR life quality OR QOL OR HRQOL OR HR-QOL OR HRQL OR holistic OR biopsychosocial OR bio-psycho-social OR person-centred OR person-centered OR patient-centred OR patient-centered OR side-effect* OR side effect* OR symptom*)
	**MEDLINE/PubMed/PsycINFO (Ovid)**
1	exp Testicular Neoplasms/
2	((testic* or testis or testes) adj3 (cancer or carcinoma or neoplas* or tumour or tumor or malignan* or oncolog*)).kw,tw.
3	(seminoma or nonseminomatous or non-seminomatous or germ cell or nsgct).kw,tw.
4	1 or 2 or 3
5	exp Qualitative Methods/ or exp Qualitative Measures/ or exp Qualitative Research/ or exp Interviews/ or exp Interview/ or exp Interview, psychological/ or exp Interview/ or exp Focus Group/ or exp Focus Groups/ or exp Ethnography/
6	(interview* or focus group* or ethnograph* or qualitative research or qualitative study or qualitative analysis or qualitative methods).kw,tw.
7	exp Quality of Life/
8	(quality of life or life quality or QOL or HRQOL or HR-QOL or HRQL or holistic or biopsychosocial or bio-psycho-social or person-centred or person-centered or patient-centred or patient-centered or side-effect* or side effect* or symptom*).kw,tw.
9	5 or 6 or 7 or 8
10	4 and 9
	**Web of Science**
1	(AB=(cancer* OR tumour OR tumor OR malignan* OR oncolog*)) AND AB=(testicular OR testis OR testes OR testicle)
2	(AB=(interview* OR focus group* OR ethnograph* OR qualitative research OR qualitative study OR qualitative analysis OR qualitative methods)) OR AB=(quality of life OR life quality OR QOL OR HRQOL OR HR-QOL OR HRQL OR holistic OR biopsychosocial OR bio-psycho-social OR person-centred OR person-centered OR patient-centred OR patient-centered OR side-effect* OR side effect* OR symptom*)
3	#1 AND #2
